# A Systematic Review and Meta-Analysis of 12-Month Patency After Intervention for Iliofemoral Obstruction Using Dedicated or Non-Dedicated Venous Stents

**DOI:** 10.1177/15266028211057085

**Published:** 2021-11-10

**Authors:** Ghulam M. Majeed, Krishan Lodhia, Jemima Carter, Jack Kingdon, Rachael I. Morris, Adam Gwozdz, Athanasios Saratzis, Prakash Saha

**Affiliations:** 1 Academic Department of Vascular Surgery, St. Thomas’ Hospital, School of Cardiovascular Medicine & Sciences, King’s College London, London, UK; 2 NIHR Leicester Biomedical Research Centre, Leicester, UK

**Keywords:** DVT, stent, thrombosis, venous occlusion, nitinol stent

## Abstract

**Background:**

Endovascular stenting of the deep venous system has been proposed as a method to treat patients with symptomatic iliofemoral outflow obstruction. The purpose of this systematic review and meta-analysis was to compare the effectiveness of this treatment at 1-year following the development of dedicated venous stents.

**Method and results:**

We searched MEDLINE and EMBASE for studies evaluating the effectiveness of venous stent placement. Data were extracted by disease pathogenesis: non-thrombotic iliac vein lesions (NIVL), acute thrombotic (DVT), or post-thrombotic syndrome (PTS). Main outcomes included technical success, stent patency at 1 year and symptom relief. A total of 49 studies reporting outcomes in 5154 patients (NIVL, 1431; DVT, 950; PTS, 2773) were included in the meta-analysis. Technical success rates were comparable among groups (97%-100%). There were no periprocedural deaths. Minor bleeding was reported in up to 5% of patients and major bleeding in 0.5% upon intervention. Transient back pain was noted in 55% of PTS patients following intervention. There was significant heterogeneity between studies reporting outcomes in PTS patients. Primary and cumulative patency at 1 year was: NIVL—96% and 100%; DVT—91% and 97%; PTS (stents above the ligament)—77% and 94%, and; PTS (stents across the ligament)—78% and 94%. There were insufficient data to compare patency outcomes of dedicated and nondedicated venous stents in patients with acute DVT. In NIVL and PTS patients, stent patency was comparable at 1 year. There was inconsistency in the use of validated tools for the measurement of symptoms before and after intervention. When reported, venous claudication, improved in 83% of PTS patients and 90% of NIVL patients, and ulcer healing occurred in 80% of PTS patients and 32% of NIVL patients.

**Conclusions:**

The first generation of dedicated venous stents perform comparably in terms of patency and clinical outcomes to non-dedicated technologies at 1 year for the treatment of patients with NIVL and PTS. However, significant heterogeneity exists between studies and standardized criteria are urgently needed to report outcomes in patients undergoing deep venous stenting.

## Introduction

Iliofemoral venous obstruction is a common condition that affects the deep veins of the pelvis and can lead to long-term disability^1-4^ that is associated with impaired quality of life.^1,5-8^ The obstruction may be caused by thrombotic (acute deep vein thrombosis [DVT] or chronic post-thrombotic scarring) or non-thrombotic pathologies, including compression from overlying structures such as the right common iliac artery, or a malignancy. Symptoms can vary and are largely dependent upon the cause, the extent of obstruction, and the duration of the disease.

Acute iliofemoral DVT usually causes severe pain, lower extremity swelling and can lead to life-threatening pulmonary embolism. In rare cases, it may also be limb-threatening.^9^ Incomplete resolution of the initial thrombus and the formation of scar tissue in the lumen of the vein more often, however, leads to chronic outflow obstruction resulting in venous hypertension and the development of post-thrombotic syndrome (PTS). Symptoms of PTS include pain, particularly on walking (venous claudication), swelling, skin changes, and in severe cases, venous ulceration.^2,10,11^ Similar signs and symptoms may also be observed in non-thrombotic causes of chronic outflow obstruction, and both are associated with a significant psychological and financial burden.^12-16^

Conservative treatment with anticoagulation and compression stockings alone may be insufficient to resolve severe symptoms, prevent recurrence,^17^ and avoid development of post-thrombotic syndrome.^18^ Therefore, in recent years, endovascular therapies have been proposed as a potential treatment for deep venous obstruction with balloon angioplasty and stenting.^19-21^ Good outcomes have been reported in large case series of patients using re-purposed arterial stents,^22-25^ however, concerns regarding complications such as stent migration,^26^ contralateral vein thrombosis caused by placement of the stent against the vessel wall of the inferior vena cava,^27,28^ and imprecise deployment systems, have led to the development of dedicated venous stent technology. A number of these devices have now become available worldwide,^29,30^ and while there have been some concerns raised about their migration and deployment mechanism leading to product recall in some instances, the use of this technology appears to be ever increasing.

A previous systematic review was carried out before the availability of these new technologies^31^ and the present study aims to see if there has been a difference in 12-month outcomes following the introduction of the first-generation of dedicated venous stents.

## Methods

### Literature Search

The study was performed according to the Preferred Reporting Items for Systematic Reviews and Meta-analyses (PRISMA).^32^ The search was limited to articles published in English. We also searched the reference lists of any systematic reviews our initial search returned to identify further literature. The Medline and Embase databases (both since inception) were searched for studies of stent placement for treatment of iliofemoral venous outflow obstruction using the following search terms: “venous” AND “stent” AND “thrombosis” NOT “coronary.” The final search was conducted on the December 12, 2020.

### Eligibility Criteria and Selection of Studies

Three researchers independently selected studies for inclusion in the review. Disagreements were resolved by consensus and discussion with the senior author. The initial inclusion criteria when screening abstracts accounted for stent location in the iliocaval tract, and studies recruiting patients with acute DVT, PTS, and non-thrombotic iliac vein lesions (NIVL). Full texts of the remaining articles were retrieved and reviewed. Studies were excluded if there was: failure to adequately define pathology, stent placement at a different site such as the portal veins and venous sinus tract, conditions not relevant to the objective such as Budd-Chiari syndrome and/or Nutcracker syndrome, reporting in select groups that is, pediatric patients, patients with malignancy and/or pregnant patients and, failure to clearly report follow up for patency rate at a defined range of 12 to 24 months with available numbers at risk. Where possible, efforts were made to reduce the reporting of duplicate patients that may have occurred by their inclusion in separate manuscripts examining different questions or when there was an overlapping timeline with previous studies reporting from the same center.

Studies were identified and classified into either non-thrombotic iliac vein lesions (NIVL), acute thrombotic (DVT) or post-thrombotic pathology (PTS). Studies that reported a mix of pathologies without reporting the data for each pathology separately were excluded.

### Data Extraction

An initial database was developed, pilot tested, and refined for subsequent use in our study. Data were extracted from peer-reviewed articles by three authors and verified by the senior author. Standardized data extraction forms were used to maintain consistency of the indications for intervention and the outcomes reported in the literature. Only data specifically involving patients receiving a stent were extracted.

### Outcome Measures

The main outcomes of interest were technical success, adverse events, stent patency at 12 months and recorded change in patients’ symptoms. Secondary patency and cumulative patency were treated as the same. Primary patency was defined as patency following the index procedure without any further intervention. Primary assisted patency was defined as patency following intervention without stent occlusion. Secondary patency was defined as patency following intervention to open an occluded stent. Patency data were extracted from the text and (where relevant/possible) Kaplan-Meier curves at annual intervals and only when the numbers at risk were evident. The severity of symptoms and quality of life based on the use of an individual scoring system were recorded when available.

### Assessment of Risk of Bias

The Institute of Health Economics of Alberta Canada’s Quality Appraisal Checklist for Case Series Studies^33^ was used for assessing the quality of the included studies and the risk of bias. The checklist assesses bias based on the following 8 categories: study objective, study design, study population, interventions and co-intervention, outcome measures, statistical analysis, results/conclusions and competing interests/sources of support. Each paper is then given a final score out of 20, with higher scores indicating a lower risk of bias.

### Data Analysis

For patient-, study-, and procedure-related data, continuous variables are reported as mean or median, and categorical variables as counts or percentages (proportions). Denominators were adjusted when appropriate to include the number of patients, limbs or procedures. Data were extracted by disease pathogenesis: acute DVT, NIVL, and PTS as well as the type of stent used: dedicated or non-dedicated venous stent technology.

Following data extraction, statistical analyses were performed using the R-package for Microsoft Windows (version 3.6). Where possible, the proportions of events per outcome of interest were combined using proportional meta-analysis (“metaprop” package). Inter-study heterogeneity was analyzed using the I^2^ statistic. An I^2^ value ≥ 50% reflects significant heterogeneity as a result of real differences in populations, protocols, interventions, and outcomes. A random effects model was used in all cases in this report, due to the degree of heterogeneity in reporting amongst the studies included. For each outcome of interest, the pooled weighted estimate and 95% confidence interval (CI) were calculated and reported; relevant forest plots were also generated. *P* values were two-sided with a significance level <.05.

## Results

### Search Results

**Figure 1.**
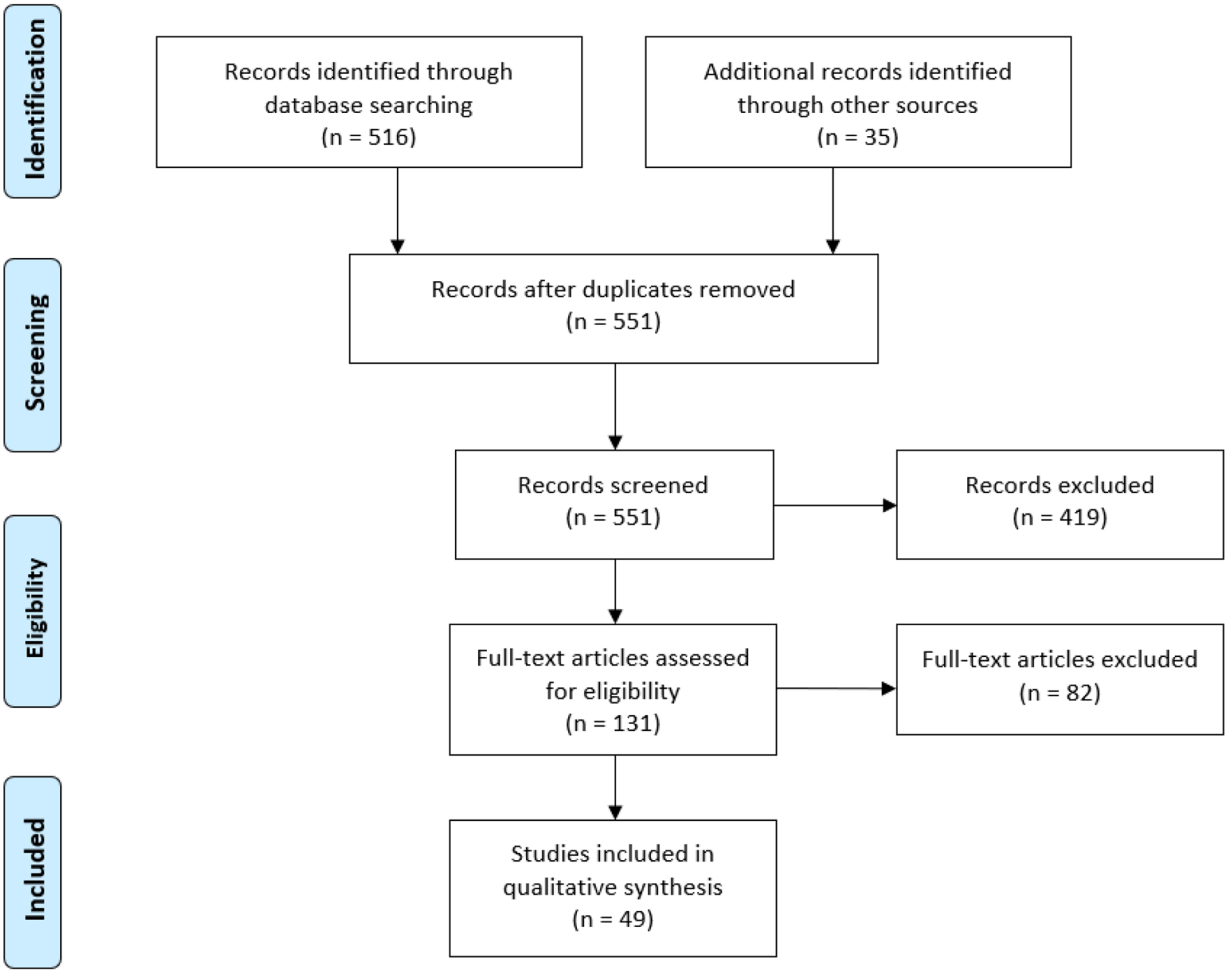
The Preferred Reporting Items for Systematic reviews and Meta-Analyses (PRISMA) flow diagram of the systematic literature review of studies reporting use of venous stents.

After screening 551 studies for eligibility, 49 studies reporting outcomes in 5154 patients (NIVL, 1431 patients; acute DVT, 950 patients; PTS, 2773 patients) between 2005 and 2020 were included in the meta-analysis. Common reasons for exclusion included stent placement outside the cava-iliofemoral venous system, failure to specify disease pathology, failure to specify stent brand used for intervention, failure to report patency rate data at a defined follow up of 12 to 24 months, and non-stenting procedures/interventions. Overall, 11 of the 49 papers included data from multiple pathologies, 9 studies reported distinct outcomes on any two of the three pathologies analyzed and 2 studies on reported all three pathologies. As these papers made a distinction between the results for each group and reported individual outcomes for each group, they were treated as separate studies in the analysis. A flow diagram of study identification and selection is shown in Figure 1.

### Study Characteristics

The majority of studies included in the meta-analysis were retrospective (Table 1). The median number of patients per study was 59 (range: 6-870) and the median number of limbs per study was 77 (range: 9-982). Median patients/limbs followed up at 1 year was 73% (range: 22-100) and the median follow-up was 22 months (range: 6-68).

**Table 1.** Characteristics of Included Studies for Analysis (*Median Values).

Study	Date of publication	Country	Study design	ROB score (/20)	Number of centers	Pathology type	Treatment period	Mean/median follow up (months)	Stent trade name	Patients Anticoagulation Adverse events	Age (Median/mean)	Limbs	Mean/median follow up (months)	% of patients/limbs followed up at one year
Kwak et al.^34^	2005	South Korea	R	12	1	DVT	2000-2004	21	Wallstent	22	58*	—	21	73
Husmann et al.^35^	2007	Switzerland	R	13	1	DVT	2000-2005	22	Wallstent	11	34*	—	22	82
Neglén et al.^22^	2007	USA	R	14	1	NIVL PTS	1997-2005	22	Wallstent	870	54*	982	22	30
Raju & Neglén^23^	2009	USA	R	13	1	PTS	1999-2007	—	Wallstent	167	53*	70	—	42
Hölper et al.^36^	2010	Germany	R	10	1	DVT	1996-2007	68*	Wallstent Palmaz	25	—	—	68*	88
Jeon et al.^37^	2010	South Korea	R	13	1	DVT	1999-2007	6*	Wallstent	30	57	—	6*	70
Rosales et al.^38^	2010	Norway	R	12	1	PTS	2000-2009	33*	Wallstent	34	41*	—	33*	62
Wahlgren et al.^39^	2010	Sweden	R	10	1	PTS	2003-2007	23	Wallstent	15	—	16	23	69
Meng et al.^40^	2011	China	R	10	1	NIVL	1997-2008	46*	Wallstent	272	—	—	46*	36
Hager et al.^41^	2013	USA	R	8	2	DVT PTS	2006-2010	26*	Wallstent Protégé	70	52*	77	26*	73
Meng et al.^42^	2013	China	P	11	1	DVT	2008-2011	11	E-Luminexx SMART	45	—	45	11	89
Alerany et al.^43^	2014	Spain	R	11	1	PTS	2009-2012	21	Wallstent	36	50	41	21	66
Liu et al.^44^	2014	China	P	15	—	NIVL PTS	2008-2012	12*	Wallstent	48	41	—	12*	49
Matsuda et al.^45^	2014	Japan	R	11	1	DVT	2000-2008	13	Wallstent E-Luminexx SMART	13	63	14	13	77
Park et al^46^	2014	South Korea	R	12	1	DVT	2005-2011	16	Wallstent SMART	51	70*	—	16	37
Sang et al.^47^	2014	China	R	11	—	PTS	2005-2012	36	E-Luminexx SMART	67	44	—	36	87
Ye et al.^48^	2014	China	R	13	1	PTS	2007-2011	25*	Wallstent E-Luminexx	110	51	118	25*	73
Zhu et al.^49^	2014	China	P	13	1	DVT	2010-2012	18	E-Luminexx	26	54	—	18	100
Fatima et al.^50^	2015	USA	R	11	1	PTS	2005-2014	10*	Wallstent Protégé SMART	28	48	—	10*	46
Park & So^51^	2015	South Korea	R	11	1	DVT	2001-2007	56	SMART	37	57	—	56	100
Srinivas et al.^52^	2015	India	P	14	1	DVT	2011-2013	12	Wallstent SMART	8	42	9	12	88
Yin et al.^53^	2015	China	R	13	1	PTS	2007-2012	21*	Wallstent Everflex ev3 endovascular	122	46*	122	21*	64
Falcoz et al.^54^	2016	France	P	11	1	PTS	2012-2016	18*	Protégé	21	41*	—	18*	100
Jia et al.^55^	2016	China	R	11	1	DVT	2010-2013	22	E-Luminexx	68	62	—	22	94
Ye et al.^56^	2016	China	R	13	1	PTS	2010-2014	27*	Wallstent E-Luminexx	24	56*	45	27*	83
Abdul-Haqq et al.^57^	2017	USA	R	11	1	NIVL DVT PTS	2003-2015	20	Wallstent	70	42	74	20	61
Engelberger et al.^58^	2017	Switzerland	P	12	1	DVT	2011-2013	9	Sinus venous Sinus XL Sinus XL superflex Zilver vena	36	49	—	9	100
Jiang et al.^59^	2017	China	P	11	1	DVT	2008-2012	24	E-Luminexx SMART	27	53	28	24	96
Murphy et al.^60^	2017	USA	R	10	—	PTS	200-2015	48	Wallstent	71	51	—	48	83
Partovi et al.^61^	2017	USA	R	10	1	PTS	2008-2010	51	Wallstent	7	55	—	51	100
Ruihua et al.^62^	2017	China	R	13	1	PTS	2013-2014	19*	Wallstent E-Luminexx	81	57	81	19*	89
van Vuuren et al.^63^	2017	Netherlands	P	14	1	NIVL PTS	2009-2016	—	Sinus obliquus Sinus venous Sinus XL Sinus XL superflex Veniti vici Venovo Zilver vena	369	43	417	—	53
Black et al.^64^	2018	UK	R	10	1	PTS	2014-2016	20*	Sinus XL Vici venovo	88	42*	101	20*	67
Grøtta et al.^65^	2018	Norway	R	11	1	PTS	2009-2016	44*	Wallstent	39	46*	39	44*	69
Liu et al.^66^	2018	China	R	12	—	DVT	2014-2016	12	Wallstent	91	56	—	12	95
Rizvi et al.^67^	2018	USA	R	11	—	NIVL	2013-2014	14*	Wallstent	210	72	268	14*	70
Ye et al.^68^	2018	China	R	12	1	PTS	2012-2015	22*	Wallstent E-Luminexx	114	49	114	22*	62
Yu et al.^69^	2018	China	R	12	—	DVT	2009-2014	38	SMART	40	57	46	38	100
Avgerinos et al.^70^	2019	USA	R	10	—	DVT	2007-2017	32	Wallstent Protégé	72	46	77	32	44
Funatsu et al.^71^	2019	Japan	R	12	10	DVT	2000-2014	21*	Wallstent E-Luminexx Epic Express Palmaz SMART	59	68	—	21*	41
Gagne et al.^25^	2019	USA	R	12	1	NIVL PTS	2007-2014	50*	Wallstent	67	63*	75	50*	87
Ignatyev et al.^72^	2019	Russia	R	9	1	NIVL PTS	2009-2017	60	Wallstent	75	42*	—	60	90
Menez et al.^73^	2019	France	R	13	1	PTS	2010-2015	21	Sinus XL Zilver vena	95	41	108	21	80
Razavi et al.^74^	2019	USA	P	13	22	NIVL PTS	2015-2016	12	Veniti Vici	170	54	171	12	74
Jayaraj et al.^75^	2020	USA	R	10	1	NIVL PTS	2014-2017	26*	Wallstent	555	—	474	26*	66
Jiang et al^76^	2020	China	R	12	1	DVT	2014-2016	24	Wallstent SMART	46	62	—	24	100
Lichtenberg et al.^77^	2020	Germany	P	12	1	NIVL PTS	2016-2017	—	Venovo	79	57	85	—	67
Moeri et al.^78^	2020	Switzerland & Germany	R	11	—	PTS	2008-2019	12*	Blueflow Sinus Repo Sinus XL Venovo Vici venous	150	46	—	12*	22
Sebastian et al.^79^	2020	Switzerland	P	15	—	NIVL DVT PTS	—	21*	Abre Sinus XL Sinus XL superflex Sinus obliquus Venovo Vici venovo Zilver vena	379	45	447	21*	84

Abbreviations: DVT = acute thrombotic; NIVL = non-thrombotic iliac vein lesions; PTS = post-thrombotic syndrome; R/P = retrospective/prospective; ROB = risk of bias.

### Assessment of Risk of Bias

Risk of bias was assessed using The Institute of Health Economics of Alberta Canada’s Quality Appraisal Checklist for Case Series Studies. Papers were awarded a score out of 20 with higher scored indicating a lower risk of bias. Scored ranged from 8 to 15. Two papers achieved a score of 15, 3 achieved a score of 14, 10 achieved a score of 13, 11 achieved a score of 12, 13 achieved a score of 11, 8 achieved a score of 10, 1 achieved a score of 9, and 1 achieved a score of 8.

### Technical Success

Technical success was high across all pathologies treated irrespective of the type of stent implanted. In patients with an acute DVT, it was 100% (95% CI: 91%-100%), in those with a non-thrombotic iliac vein lesion (NIVL) it was 100%, and in post-thrombotic syndrome (PTS) patients it was 97% (95% CI: 93%-98%).

### Stent Patency in All Groups

Patency was typically evaluated by ultrasonography, but a formal definition was rarely provided, and an independent assessment of imaging quality and findings was not carried out. Only patients with a minimum follow-up of 1 year were included in the analysis. In patients with a venous stent placed for a non-thrombotic iliac vein lesion (NIVL), the primary patency was 96% (95% CI: 94%-98%); primary-assisted patency was 100%, and secondary patency was 100% (95% CI: 75%-100%) at 1 year (Supplementary Figure 1). In patients with a venous stent placed as part of treatment for an acute DVT, the primary patency was 91% (95% CI: 88%-93%); primary assisted patency was 97% (95% CI: 93%-99%), and secondary patency was 97% (95% CI: 93%-99%) at 1 year (Supplementary Figure 2). We divided post-thrombotic syndrome (PTS) patients into studies that included patients with venous stents ending above the inguinal ligament and those who had a stent placed across it. In studies of PTS patients with stents above the inguinal ligament, the primary patency rates were 77% (95%CI: 69%-83%) at 1 year while in those with a stent placed across the ligament, the 1-year primary patency was 78% (95% CI: 73%-82%) (Supplementary Figures 3 & 4). In studies including PTS patients with stents above the inguinal ligament, the primary assisted patency rates were 83% (95% CI: 76%-89%) at 1 year while in those with a stent placed across the ligament, the one-year primary assisted patency was 88% (95% CI: 83%-92%) (Supplementary Figures 3 & 4). The secondary patency of stents placed above the ligament in PTS patients was 94% (95% CI: 88%-97%) at 1 year and in those placed across the ligament it was also 94% (95% CI: 91%-97%) at the same time point (Supplementary Figures 3 & 4). There was, however, significant heterogeneity in the PTS studies (*p* < 0.01).

### First Generation Dedicated Venous Stents Versus Non-Dedicated Venous Stent Patency

**Figure 2.**
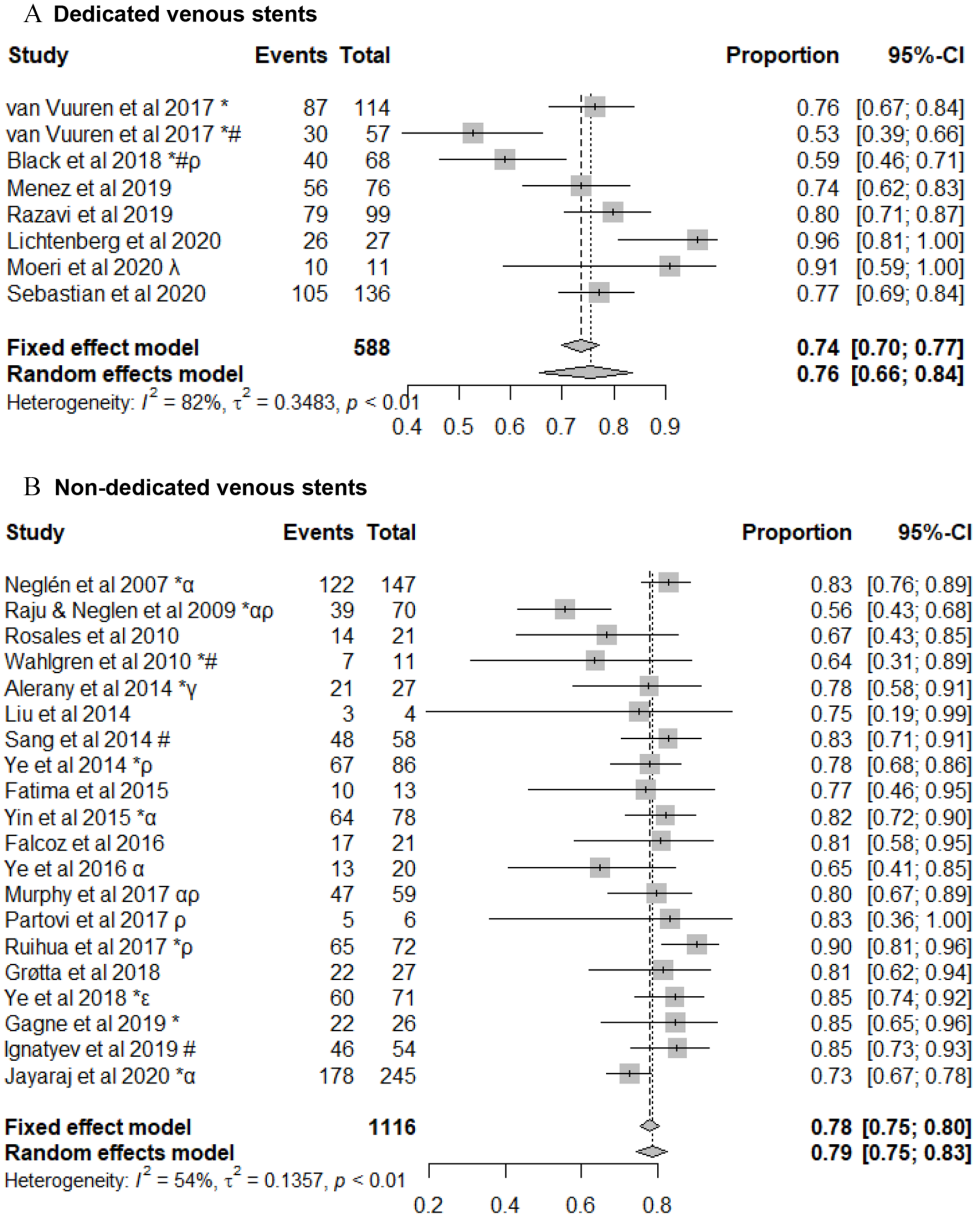
Forest plot of primary patency following stenting for treatment of post-thrombotic syndrome using (A) dedicated and (B) non-dedicated venous stents. Data are shown in descending order by year of publication with proportions of events reported. CI, confidence interval. ^*^Events reported per limb. ^α^May include some patient duplicates. ^ρ^Chronic total occlusions only. ^γ^Inclusion of 3 patients with dedicated venous stents. ^ε^Femoral vein intervention with angioplasty +/− stent carried out. ^λ^Only braided nitinol stents included from this manuscript. ^#^Inclusion of patients with endophlebectomy +/− fistula.

**Figure 3.**
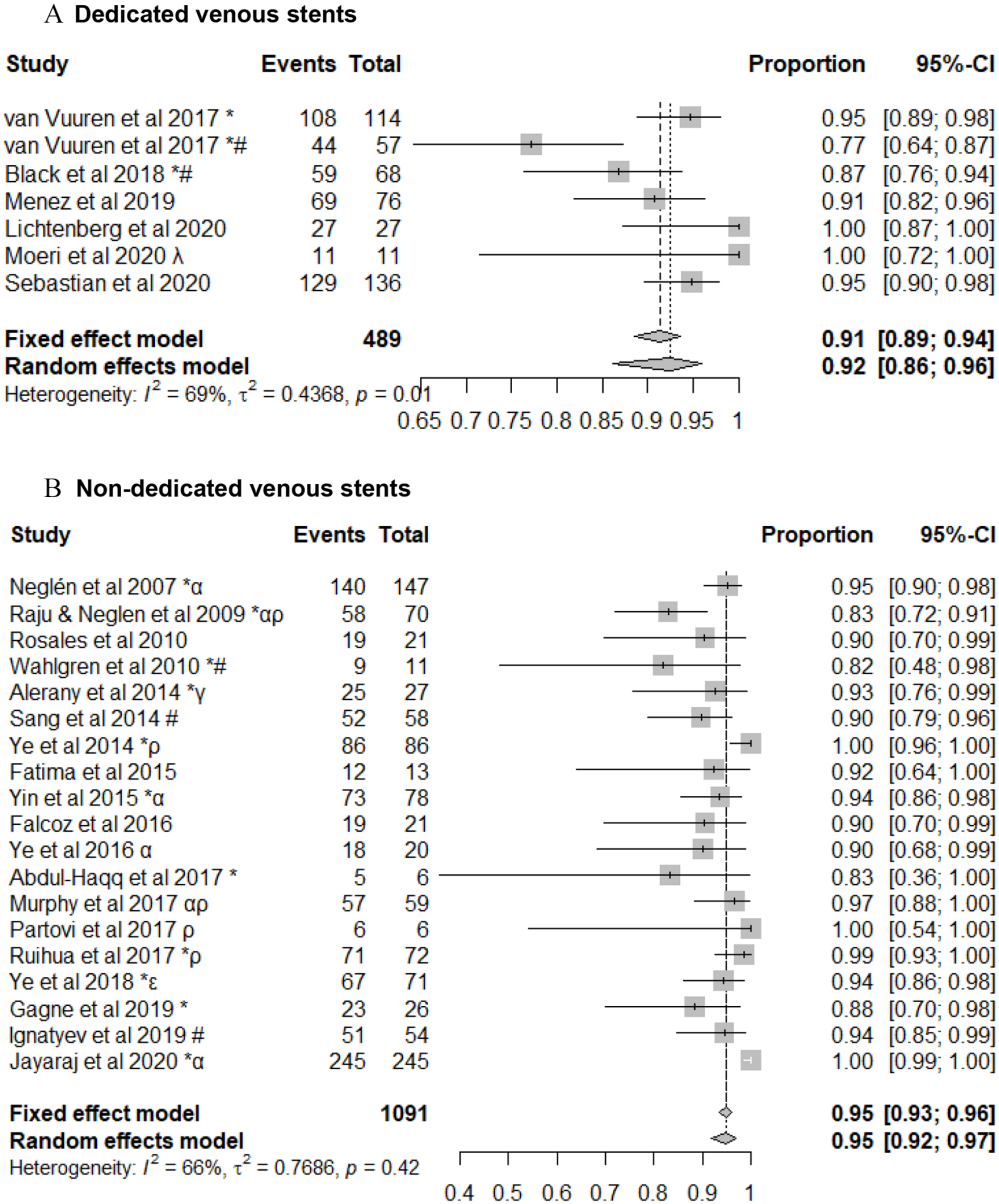
Secondary patency following stenting for treatment of post-thrombotic syndrome using (A) dedicated and (B) non-dedicated venous stents. Data are shown in descending order by year of publication with proportions of events reported. CI, confidence interval. ^*^Events reported per limb. ^α^May include some patient duplicates. ^ρ^Chronic total occlusions only. ^γ^Inclusion of 3 patients with dedicated venous stents. ^ε^Femoral vein intervention with angioplasty +/− stent carried out. ^λ^Only braided nitinol stents included from this manuscript. ^#^Inclusion of patients with endophlebectomy +/− fistula.

There was insufficient data to compare the use of first-generation dedicated venous stents with non-dedicated venous stents in patients with acute DVT. In patients with a NIVL, the primary patency, primary-assisted and secondary patencies at 1 year in patients with a dedicated venous stent were 95% (95% CI: 89%-98%); 100%, and; 100%. This was comparable to patients who were treated with non-dedicated venous stents where the primary, primary assisted and secondary patencies at 1 year were 97% (95% CI: 94%-98%); 100%, and 100% (95% CI: 86%-100%). In patients with PTS treated with a dedicated venous stent, the primary, primary assisted and secondary patencies at 1 year were 76% (95% CI: 66%-84%), 80% (95% CI: 76%-84%), and 92% (95% CI: 86%-96%) (Figures 2 & 3). This was comparable to non-dedicated stents, where the primary, primary assisted and secondary patencies at 1 year were 79% (95% CI: 75%-83%), 91% (95% CI: 85%-94%), and 95% (95% CI: 92%-97%) (Figures 2 & 3). There was, however, significant heterogeneity in the studies reporting on both first-generation venous stents and non-dedicated technology (*p* < 0.01).

### Symptom Relief

**Figure 4.**
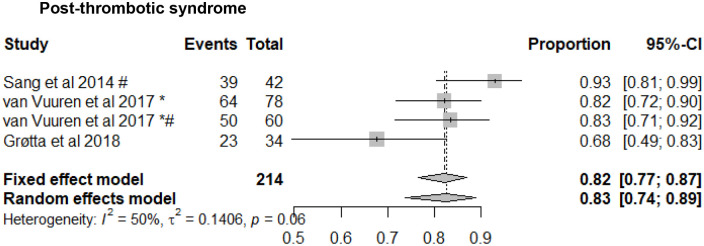
Improvement in venous claudication rates following venous stenting for post-thrombotic syndrome. Data are shown in descending order by year of publication with proportions of events reported. CI, confidence interval. ^*^Events reported per limb. ^#^Inclusion of patients with endophlebectomy +/- fistula.

**Figure 5.**
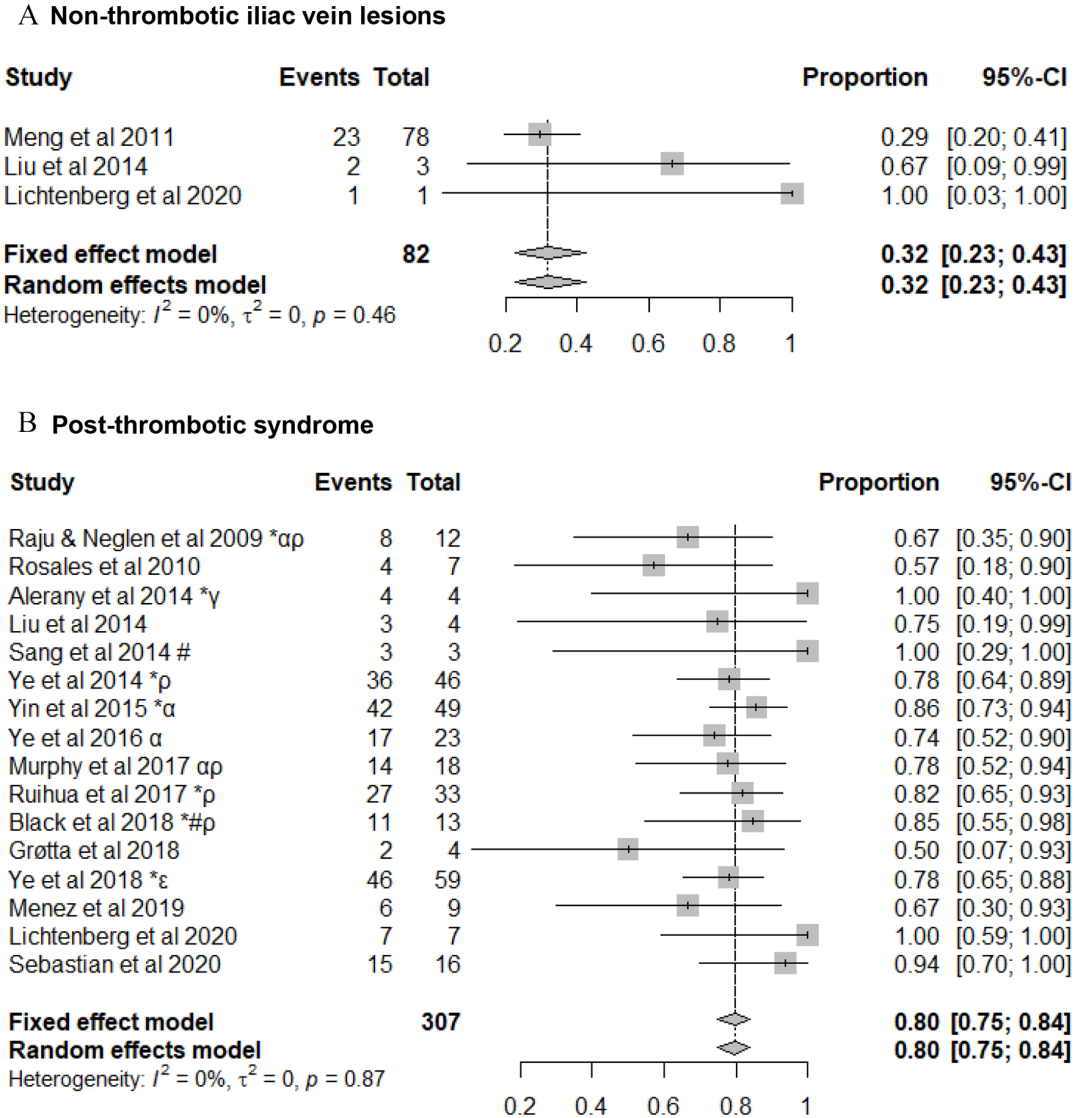
Ulcer healing rates following venous stenting for (A) non-thrombotic iliac vein lesions and (B) post-thrombotic syndrome. Data are shown in descending order by year of publication with proportions of events reported. CI, confidence interval. ^*^Events reported per limb. ^α^May include some patient duplicates. ^ρ^Chronic total occlusions only. ^γ^Inclusion of 3 patients with dedicated venous stents. ^ε^Femoral vein intervention with angioplasty +/− stent carried out. ^#^Inclusion of patients with endophlebectomy +/− fistula.

Data regarding changes in venous related symptoms following stent placement was not reported in the majority of studies. Similarly, there were inconsistencies regarding the use of objective reporting tools for the assessment of patients with chronic venous insufficiency. This made any meaningful comparisons using these methods impossible. Where data were available: venous claudication, reported in 3 papers improved in 83% (95% CI: 74%-89%) of PTS patients (Figure 4), and ulcer healing, reported in 17 studies; occurred in 80% (95% CI: 75%-84%) of PTS patients, but only 32% (95% CI: 23%-43%) of NIVL patients (Figure 5).

### Adverse Events and Side-Effects

The adverse events reported from each study included are shown in Supplementary Figure 5. There were no deaths reported in any of the studies. In patients with a venous stent placed as part of treatment for an acute DVT, minor bleeding occurred in 4% of patients (reported by 10 studies in 608 patients) and major bleeding in 0.8% of patients (reported by 7 studies in 344 patients). In patients with a venous stent placed as part of treatment for a non-thrombotic iliac vein lesion (NIVL), minor bleeding occurred in 5% of patients (reported by 3 studies in 151 patients) and major bleeding in 0.4% of patients (reported by 4 studies in 1535 patients). In patients with a venous stent placed as part of treatment for post-thrombotic syndrome (PTS), minor bleeding occurred in 4.4% of patients (reported by 11 studies in 1085 patients) and major bleeding occurred in 0.9% of patients (reported by 8 studies in 781 patients). Transient back pain was also recorded in the PTS group in 55% of patients (reported by 6 studies in 508 patients).

### Anticoagulation Use

Anticoagulation was reported in 33 of the 50 studies (Supplementary figure 6). Warfarin was prescribed to patients in 32 studies, DOACs in 7 studies, and LMWH in 4 studies. Anticoagulation was prescribed for at least 3 months by 35 of the 50 studies, the remaining papers did not report the length of time patients were anticoagulated for. Patients were anticoagulated for up to 6 months in 24 studies, up to 12 months in 7 studies and for life in 4 studies. Reporting of use of multi-disciplinary teams including hematology for these decisions were variable.

## Discussion

Here we carry out a meta-analysis of 1-year outcomes following deep venous stenting for obstruction along the iliofemoral venous outflow tract. Overall results of this analysis indicate that venous stent placement has a high technical success rate with a low risk of complications. Over half of patients having venous stent placement for post-thrombotic syndrome (PTS) report transient back pain, and this should be discussed during counseling of this procedure. Venous stents appear effective at restoring luminal flow in the majority of patients at 1 year, even if they cross the inguinal ligament, but challenges still remain at maintaining primary patency especially in PTS patients. Nevertheless, they appear to improve patient symptoms at 1 year with PTS patients reporting improvements in venous claudication and ulcer healing.

The first generation of dedicated venous stents were developed to overcome specific complications of existing technologies including stent migration, stent compression, kinking, and contralateral vein thrombosis due to “jailing” of the contralateral outflow tract. They were designed to be easier to deploy, have sufficient flexibility to follow the curve of the iliac vein and adequate radial resistive strength and crush resistance to withstand forces from an overlying iliac arterial pulsation, the compression points at the pubic rami and inguinal ligament,^80^ and the recoil of fibrotic post-thrombotic tissue. Several different dedicated venous stents are now commercially available worldwide. Perhaps surprisingly, however, the newer dedicated venous stents do not seem to outperform older technology that had been used off-label in studies that we were able to adequately analyze. Primary and secondary patency similar at one year in patients with NIVL and PTS. Whether the loss of stent patency is due to similar factors remains uncertain, however, as many studies inadequately reported their outcomes making it impossible to carry our subgroup analyses to identify the influence of any specific study- and/or patient-related characteristics. Differences in the rationale, suitability and indication for stent placement were evident and there was variability on the methods used to define a significant obstruction. In addition, changing interventional techniques; use of intravascular ultrasound; differences in post-operative management in terms of surveillance and the types and duration of anticoagulation and/or antiplatelets, and; inconsistent approaches to reintervention following the index procedure makes direct comparison between studies challenging. These differences may, in part, explain the significant heterogeneity in patency outcomes for stents placed in patients across the studies included in our analyses. Nevertheless, given the high technical success of venous stenting, urgent research is now needed to understand the factors that lead to loss of stent patency. This includes finding a better method to determine inflow pre-operatively that could improve patient selection.

Our review highlights that there is a paucity of robust, high quality, level 1 evidence to support the use of venous stenting and there is significant reporting bias. Ongoing studies such as CLEAR-DVT are trying to provide the foundation for larger prospective randomized control trials in patients with acute DVT, while the results of C-TRACT, which aims to determine if endovascular therapy can benefit patients with PTS, are eagerly awaited. Inconsistent reporting of clinical outcomes has, however, highlighted an urgent need to develop a core outcome set to evaluate this procedure. In addition, an agreed disease specific tool to objectively assess the burden of disease on patients, especially in those with PTS, is required. The Villalta scale was recommended by the International Society for Thrombosis and Haemostasis (ISTH) for use in clinical trials but since its conception, the scoring system has been inconsistently modified. It is based on subjective criteria, which limits its precision, and it has been criticized for not being disease-specific.^81^ Qualitative studies of patient experience and expert opinion suggests that the Villalta score may also fail to capture typical PTS complaints or their importance to patients,^82^ and a major drawback of this tool is that it does not include an assessment of a patient’s quality of life.^83^ This may explain why it was infrequently used in venous stent studies to date. A more appropriate, sensitive and specific gold-standard assessment measure, which incorporates patient reported outcomes, should be developed for future applied health research in this area.

The use of dedicated venous stent technology may be questioned based on these data. We were, however, unable to appropriately analyze whether adjunctive techniques are required to facilitate satisfactory outcomes when using non-dedicated technology. Use of a Z-stent placed more caudally has, for example, been recommended to mitigate against contralateral DVT.^27^ Operator technique and experience is also likely to influence outcomes and complimentary tools, such as the use of intravascular ultrasound have been proposed as a method to identify a suitable landing zone for the stent, but whether they have improved outcomes remains uncertain. Dedicated venous stents are, however, easier to deploy and are available in sizes more appropriate for use in the iliofemoral venous system thereby minimizing the need for multiple stents. Their ease of use may even be one of the reasons that there has been a recent increase in the numbers of different centers reporting their use. Further developments are, however, needed especially in the treatment of patients with PTS. Drug-eluting technologies either with drug-coated balloons or drug-eluting stents are likely to be developed in the future but further research is first needed to identify which mechanisms drive in-stent stenosis in the veins, which could then be targeted. Innovative tools are also required for reinterventions to prevent the need for repeated venoplasty and/or stent re-lining. Given the young age of patients that are being treated, maintaining stent patency will likely become a bigger challenge in the future.

### Limitations

Several limitations exist in our study. Many of the studies included in the meta-analysis were single-site, retrospective, level 4 studies and reporting was inconsistent. We were unable to analyze the data by the specific type of stent used due to small numbers and it is possible that certain types of stent design may perform better than others. Stent patency was largely based on ultrasonography and there are no agreed criteria of how to assess a venous stent using this method. Assessment of stent patency with this imaging modality can also be variable and is user dependent. In addition, the threshold for reintervention was not always defined, and when it was, it was variable between studies. The follow-up is also modest across the majority of the literature in this clinical area. Many patients that undergo deep venous interventions are younger than those having arterial interventions from which stent technology is based. Any stent that has been placed will need to function for many more years, and often for decades and longer-term outcomes are required before a true assessment of their effectiveness can be established.

## Conclusion

Venous stent placement for iliofemoral venous outflow obstruction has a high rate of technical success and satisfactory 1 year patency outcomes. Improvement in clinical symptoms and quality of life can be achieved, but they are inconsistently reported in the literature and specific patient reported outcome measures are required to improve future applied health research in this area. In addition, agreed inclusion criteria for venous stenting are still urgently needed. Finally, a detailed classification of patient pathology should be used to facilitate a more accurate comparison of patient outcomes between studies and the types of interventions that have been carried out. From the available data, the first generation of dedicated venous stents have comparable performance to non-dedicated technologies in patients with NIVL and PTS but the length of follow-up is modest and longer-term data are needed to evaluate their true effectiveness. Outcomes for patients with post-thrombotic disease are inferior to those treated for non-thrombotic or acute thrombotic disease, but irrespective of stent type, a better understanding of factors that lead to loss of patency is required.

## Supplemental Material

sj-pptx-1-jet-10.1177_15266028211057085 – Supplemental material for A Systematic Review and Meta-Analysis of 12-Month Patency After Intervention for Iliofemoral Obstruction Using Dedicated or Non-Dedicated Venous Stents Click here for additional data file. Supplemental material, sj-pptx-1-jet-10.1177_15266028211057085 for A Systematic Review and Meta-Analysis of 12-Month Patency After Intervention for Iliofemoral Obstruction Using Dedicated or Non-Dedicated Venous Stents by Ghulam M. Majeed, Krishan Lodhia, Jemima Carter, Jack Kingdon, Rachael I. Morris, Adam Gwozdz, Athanasios Saratzis and Prakash Saha in Journal of Endovascular Therapy
