# Supplementary material for: A Systematic Review and Meta-Analysis of 12-Month Patency After Intervention for Iliofemoral Obstruction Using Dedicated or Non-Dedicated Venous Stents
Source: J Endovasc Ther. 2021 Nov 10;29(3):478–92. doi: 10.1177/15266028211057085 (PMC9096580; doi:10.1177/15266028211057085)
Supplement: sj-pptx-1-jet-10.1177_15266028211057085 – Supplemental material for A Systematic Review and Meta-Analysis of 12-Month Patency After Intervention for Iliofemoral Obstruction Using Dedicated or Non-Dedicated Venous Stents [file sj-pptx-1-jet-10.1177_15266028211057085.pptx]

## Slide 1
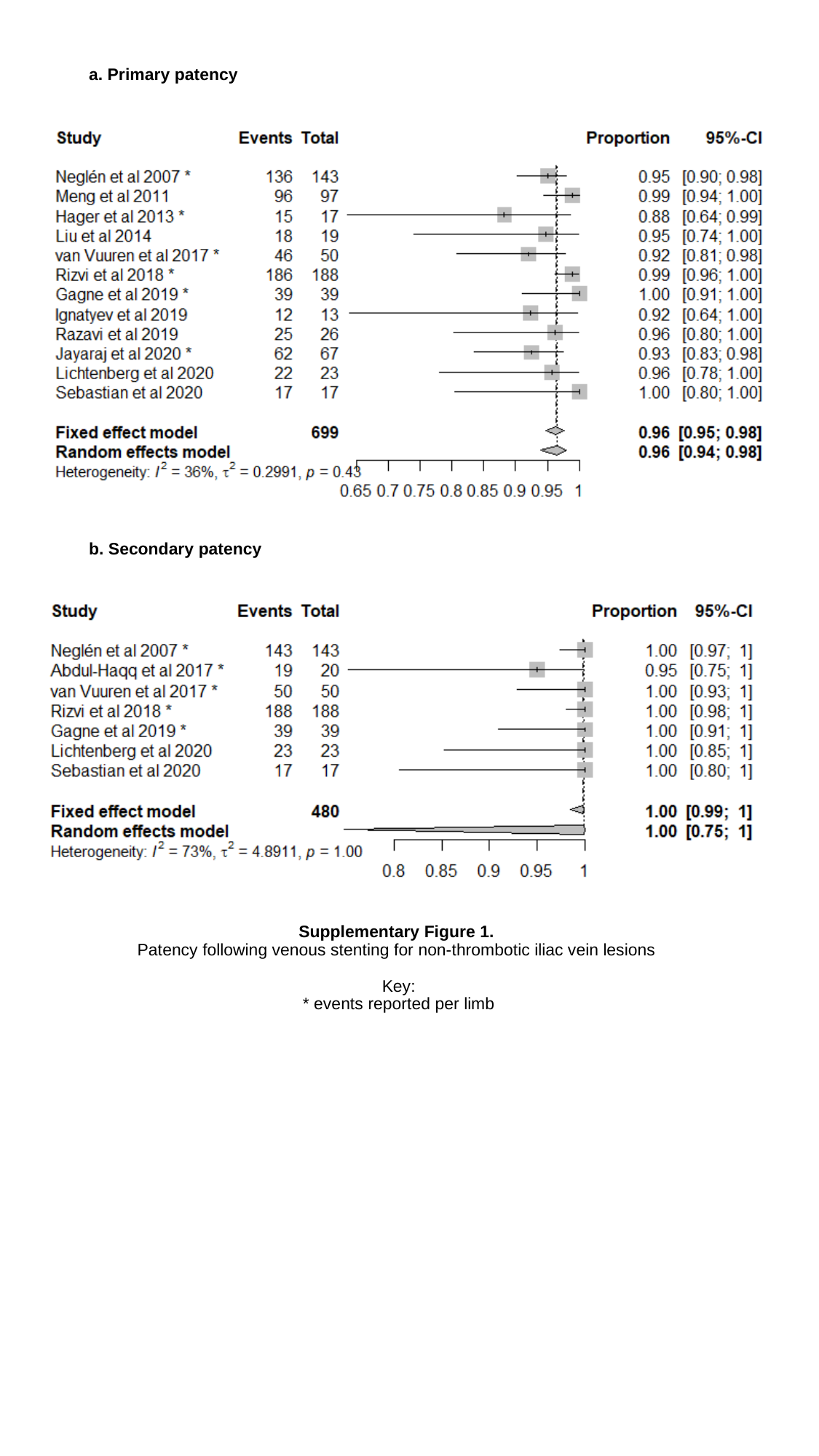

a. Primary patency
b. Secondary patency
# Supplementary Figure 1. Patency following venous stenting for non-thrombotic iliac vein lesions Key: * events reported per limb

## Slide 2
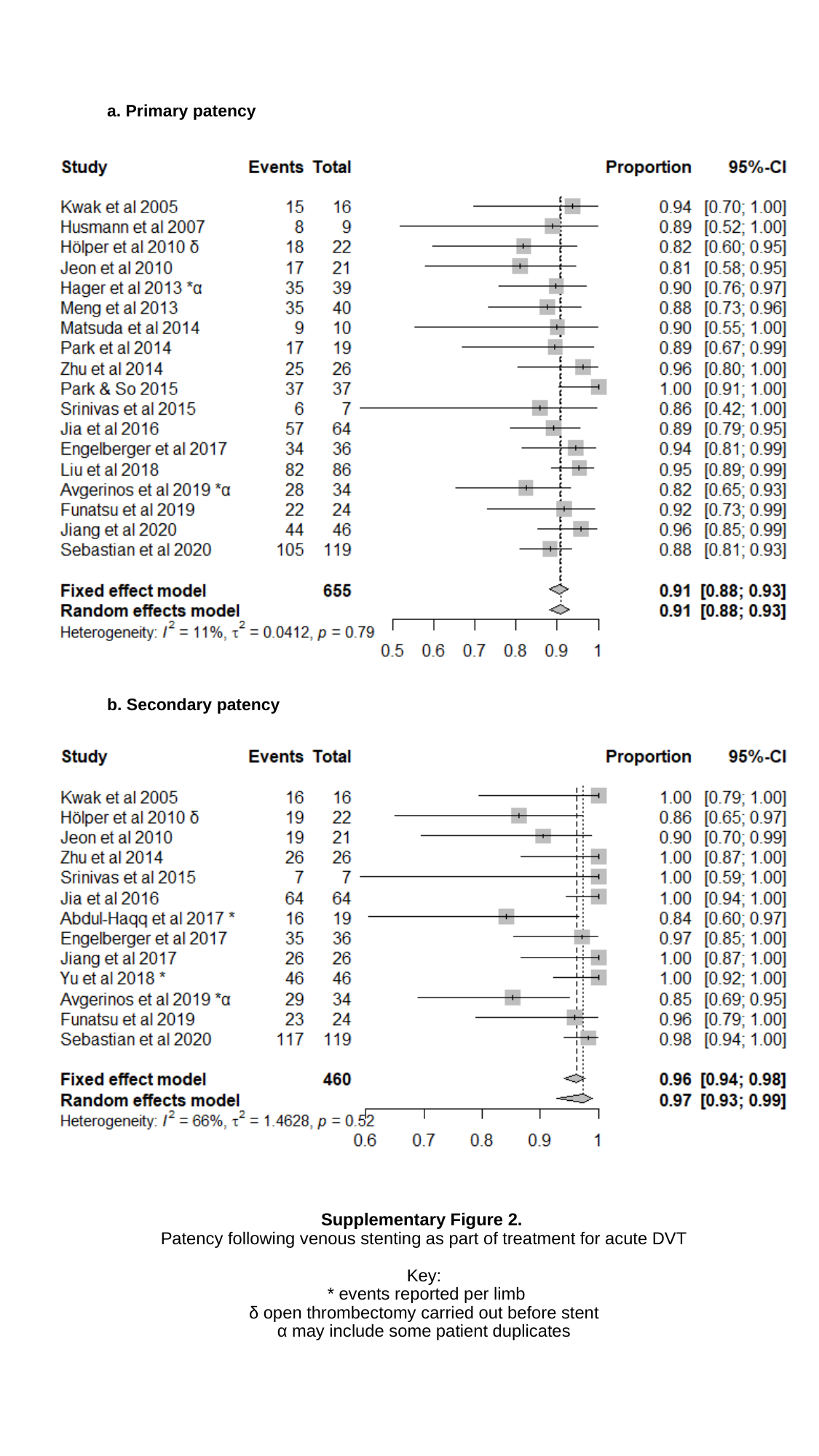

a. Primary patency
b. Secondary patency
# Supplementary Figure 2. Patency following venous stenting as part of treatment for acute DVT Key: * events reported per limb δ open thrombectomy carried out before stent α may include some patient duplicates

## Slide 3
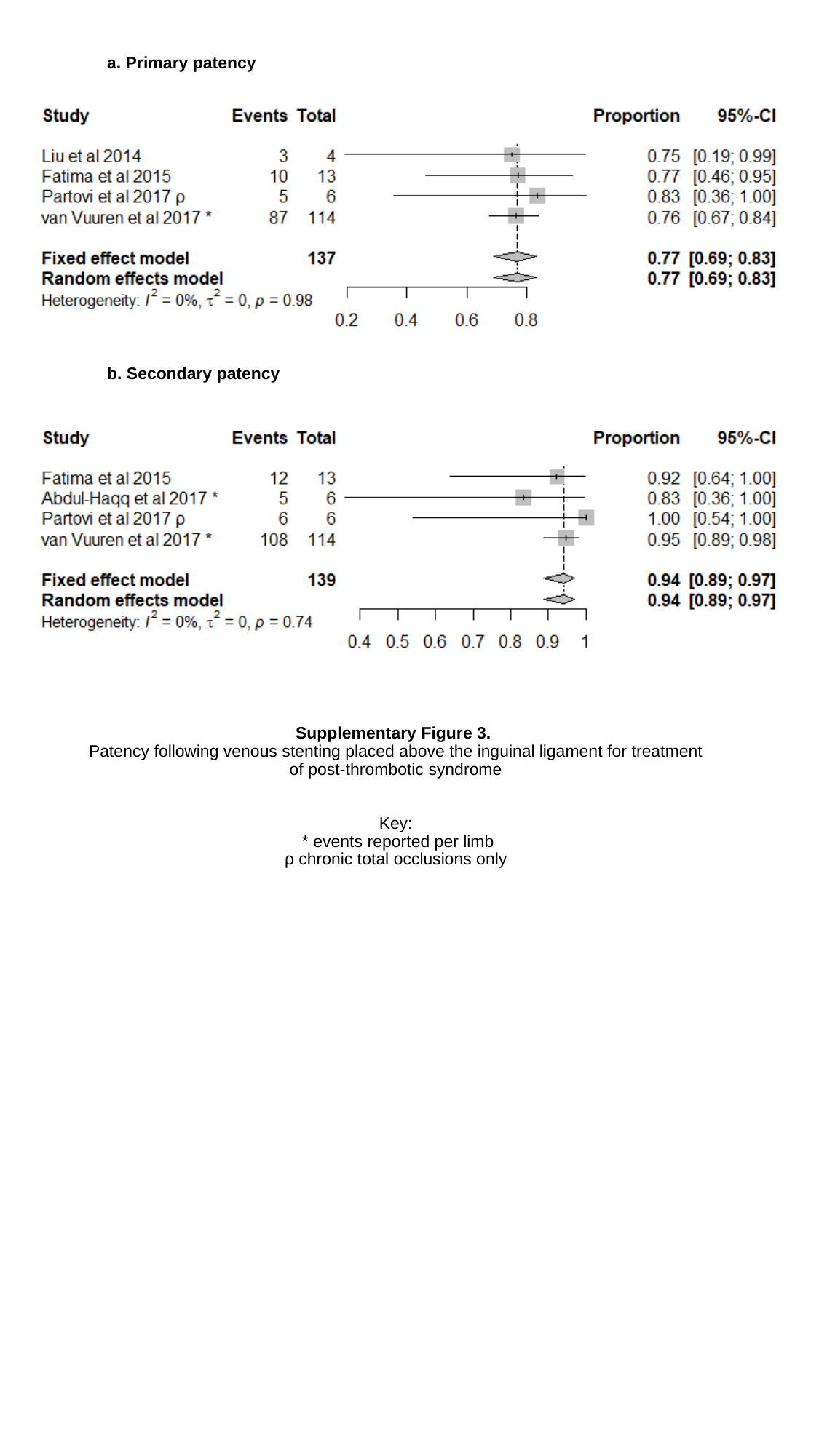

a. Primary patency
b. Secondary patency
# Supplementary Figure 3. Patency following venous stenting placed above the inguinal ligament for treatment of post-thrombotic syndrome Key: * events reported per limb ρ chronic total occlusions only

## Slide 4
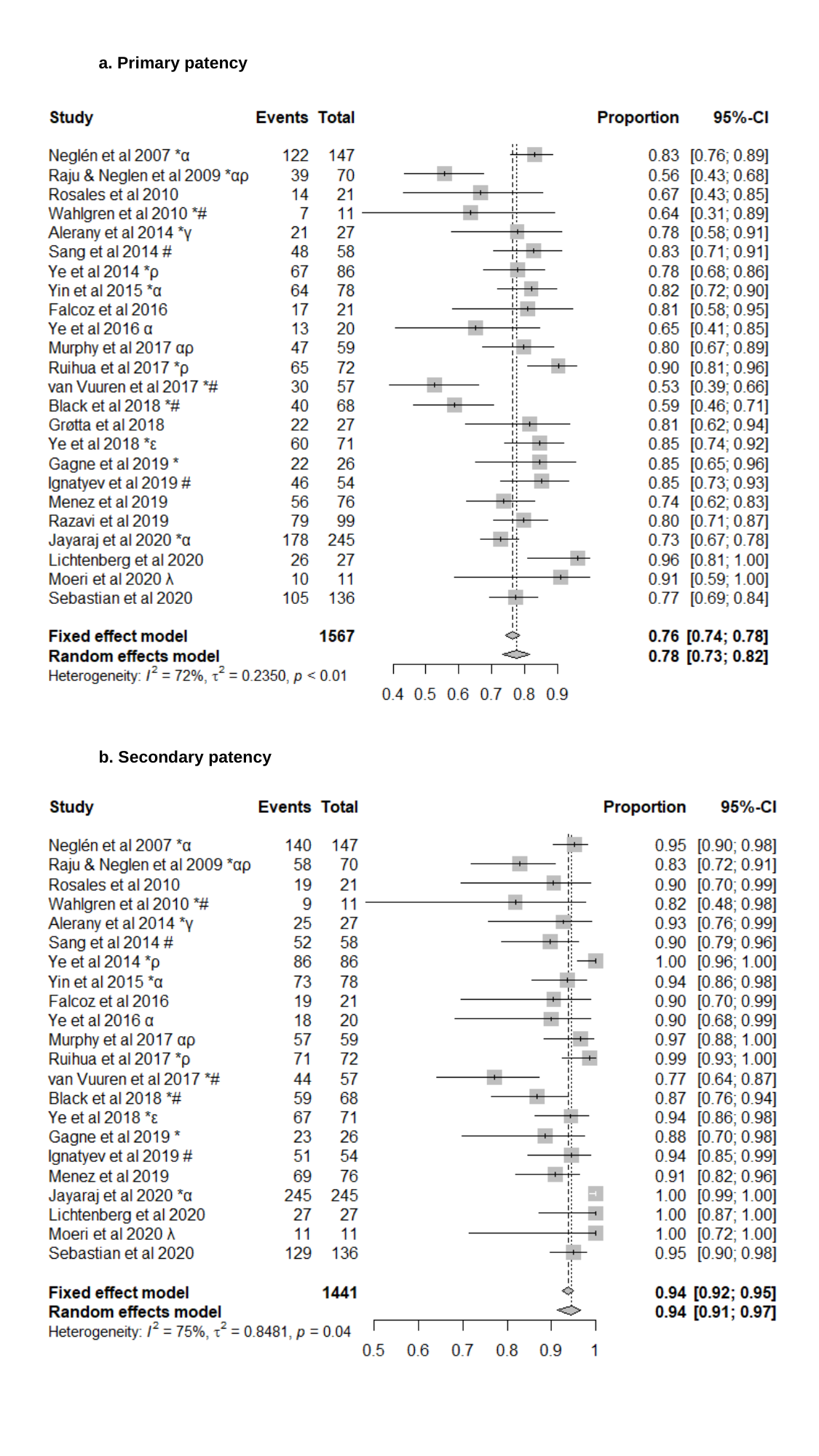

a. Primary patency
b. Secondary patency

## Slide 5
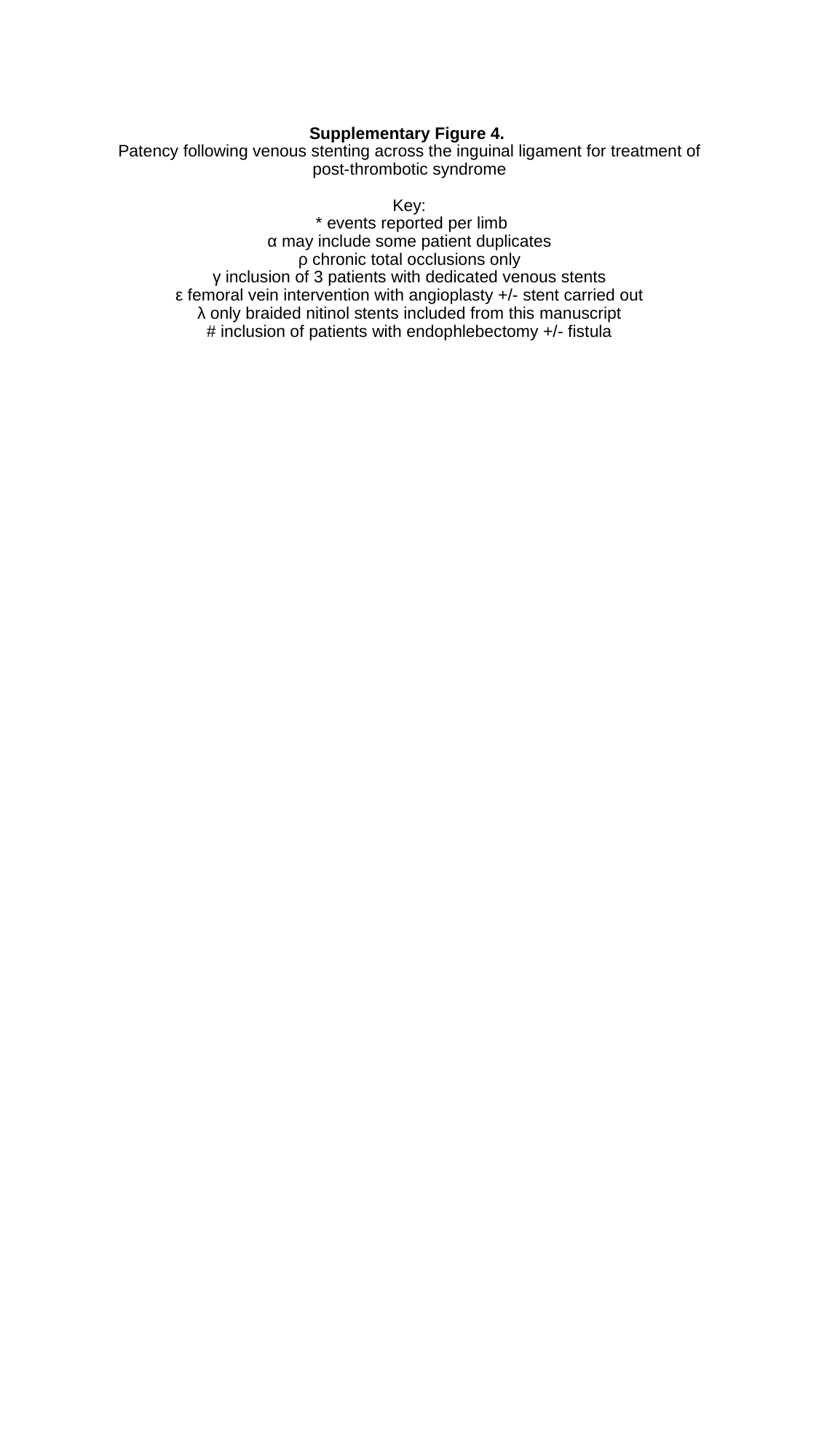

# Supplementary Figure 4. Patency following venous stenting across the inguinal ligament for treatment of post-thrombotic syndrome Key: * events reported per limb α may include some patient duplicates ρ chronic total occlusions only γ inclusion of 3 patients with dedicated venous stents ε femoral vein intervention with angioplasty +/- stent carried out λ only braided nitinol stents included from this manuscript # inclusion of patients with endophlebectomy +/- fistula

## Slide 6
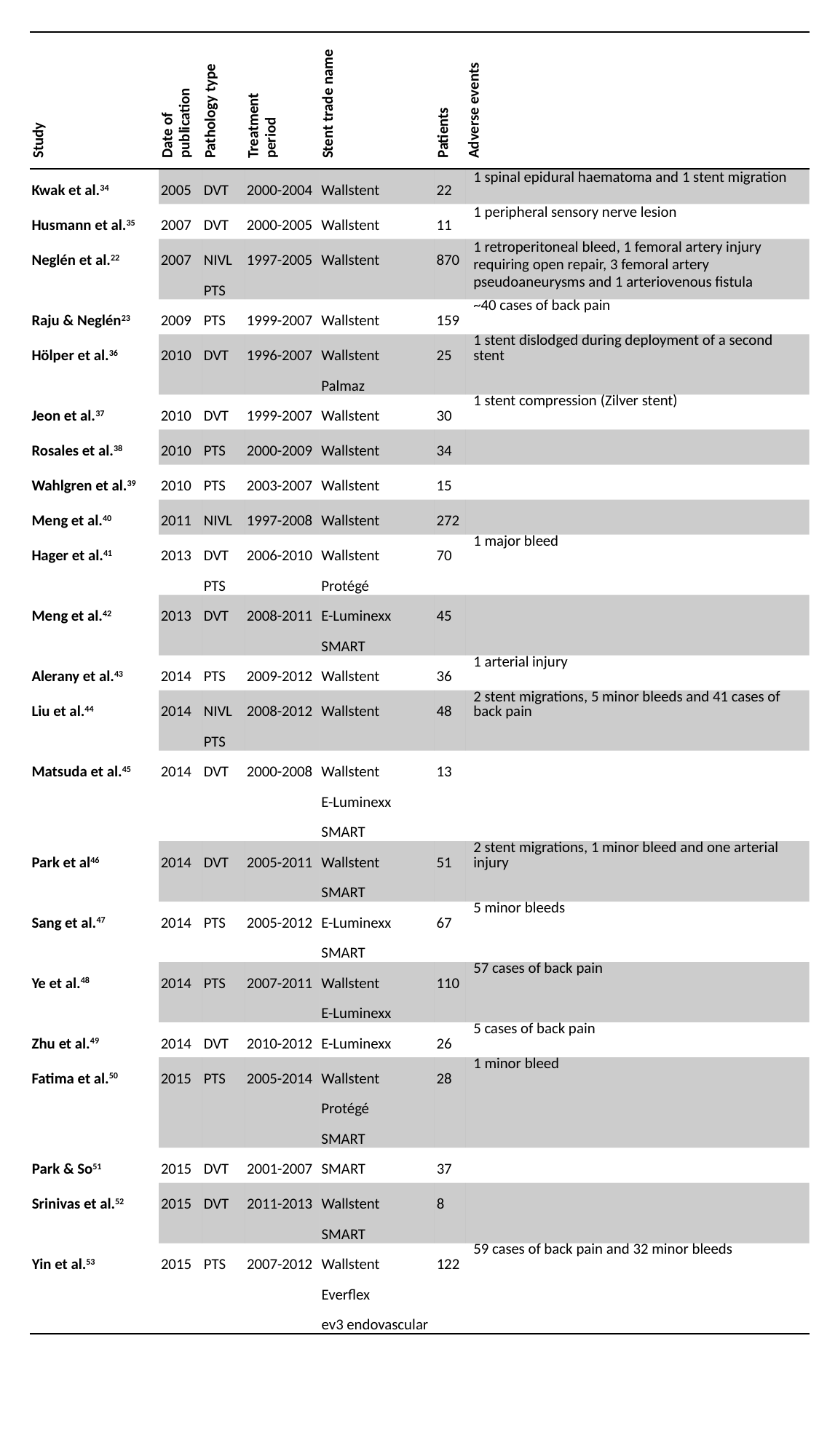

| Study | Date of publication | Pathology type | Treatment period | Stent trade name | Patients | Adverse events |
| --- | --- | --- | --- | --- | --- | --- |
| Kwak et al.34 | 2005 | DVT | 2000-2004 | Wallstent | 22 | 1 spinal epidural haematoma and 1 stent migration |
| Husmann et al.35 | 2007 | DVT | 2000-2005 | Wallstent | 11 | 1 peripheral sensory nerve lesion |
| Neglén et al.22 | 2007 | NIVL PTS | 1997-2005 | Wallstent | 870 | 1 retroperitoneal bleed, 1 femoral artery injury requiring open repair, 3 femoral artery pseudoaneurysms and 1 arteriovenous fistula |
| Raju & Neglén23 | 2009 | PTS | 1999-2007 | Wallstent | 159 | ~40 cases of back pain |
| Hölper et al.36 | 2010 | DVT | 1996-2007 | Wallstent Palmaz | 25 | 1 stent dislodged during deployment of a second stent |
| Jeon et al.37 | 2010 | DVT | 1999-2007 | Wallstent | 30 | 1 stent compression (Zilver stent) |
| Rosales et al.38 | 2010 | PTS | 2000-2009 | Wallstent | 34 | |
| Wahlgren et al.39 | 2010 | PTS | 2003-2007 | Wallstent | 15 | |
| Meng et al.40 | 2011 | NIVL | 1997-2008 | Wallstent | 272 | |
| Hager et al.41 | 2013 | DVT PTS | 2006-2010 | Wallstent Protégé | 70 | 1 major bleed |
| Meng et al.42 | 2013 | DVT | 2008-2011 | E-Luminexx SMART | 45 | |
| Alerany et al.43 | 2014 | PTS | 2009-2012 | Wallstent | 36 | 1 arterial injury |
| Liu et al.44 | 2014 | NIVL PTS | 2008-2012 | Wallstent | 48 | 2 stent migrations, 5 minor bleeds and 41 cases of back pain |
| Matsuda et al.45 | 2014 | DVT | 2000-2008 | Wallstent E-Luminexx SMART | 13 | |
| Park et al46 | 2014 | DVT | 2005-2011 | Wallstent SMART | 51 | 2 stent migrations, 1 minor bleed and one arterial injury |
| Sang et al.47 | 2014 | PTS | 2005-2012 | E-Luminexx SMART | 67 | 5 minor bleeds |
| Ye et al.48 | 2014 | PTS | 2007-2011 | Wallstent E-Luminexx | 110 | 57 cases of back pain |
| Zhu et al.49 | 2014 | DVT | 2010-2012 | E-Luminexx | 26 | 5 cases of back pain |
| Fatima et al.50 | 2015 | PTS | 2005-2014 | Wallstent Protégé SMART | 28 | 1 minor bleed |
| Park & So51 | 2015 | DVT | 2001-2007 | SMART | 37 | |
| Srinivas et al.52 | 2015 | DVT | 2011-2013 | Wallstent SMART | 8 | |
| Yin et al.53 | 2015 | PTS | 2007-2012 | Wallstent Everflex ev3 endovascular | 122 | 59 cases of back pain and 32 minor bleeds |

## Slide 7
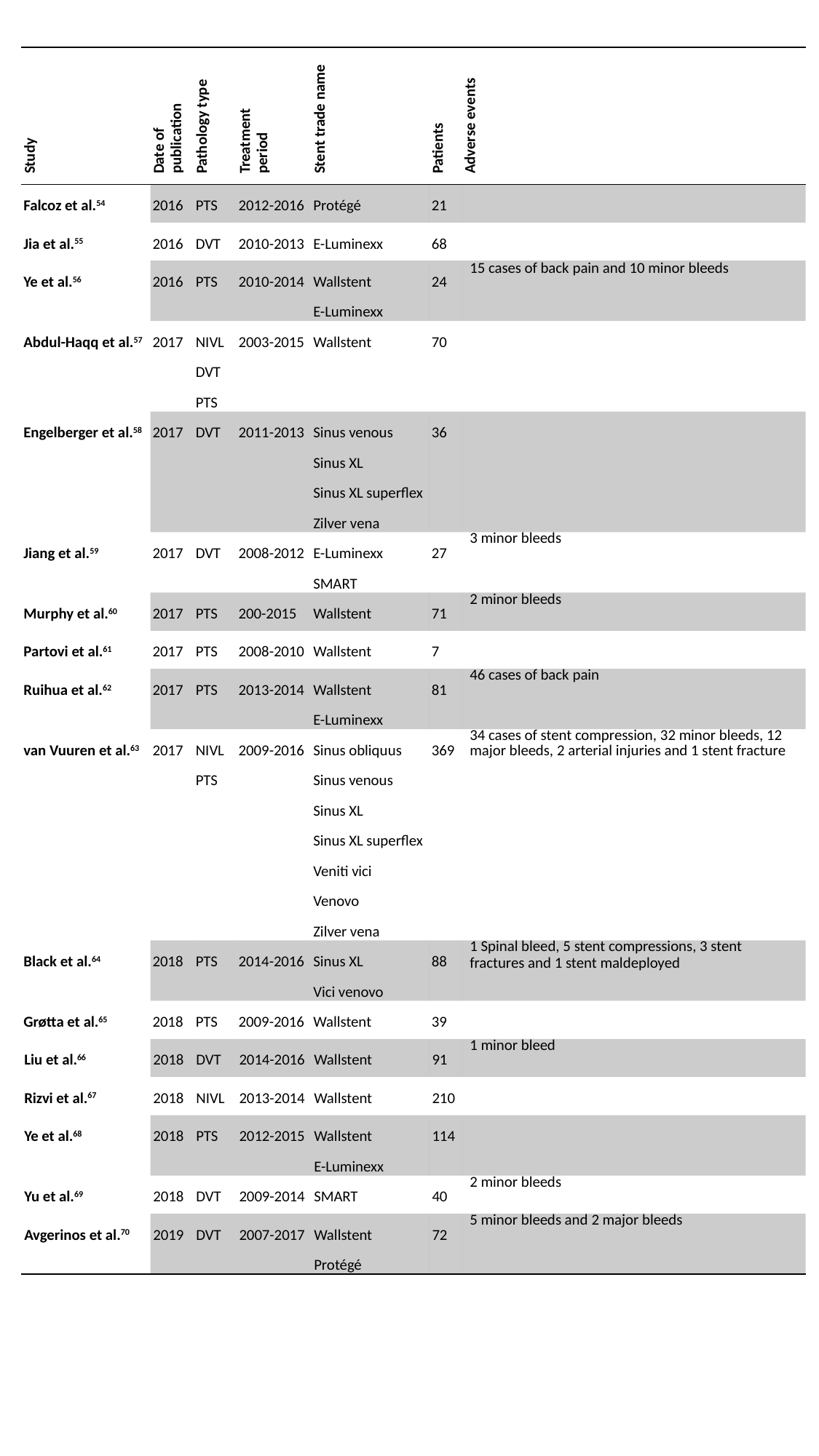

| Study | Date of publication | Pathology type | Treatment period | Stent trade name | Patients | Adverse events |
| --- | --- | --- | --- | --- | --- | --- |
| Falcoz et al.54 | 2016 | PTS | 2012-2016 | Protégé | 21 | |
| Jia et al.55 | 2016 | DVT | 2010-2013 | E-Luminexx | 68 | |
| Ye et al.56 | 2016 | PTS | 2010-2014 | Wallstent E-Luminexx | 24 | 15 cases of back pain and 10 minor bleeds |
| Abdul-Haqq et al.57 | 2017 | NIVL DVT PTS | 2003-2015 | Wallstent | 70 | |
| Engelberger et al.58 | 2017 | DVT | 2011-2013 | Sinus venous Sinus XL Sinus XL superflex Zilver vena | 36 | |
| Jiang et al.59 | 2017 | DVT | 2008-2012 | E-Luminexx SMART | 27 | 3 minor bleeds |
| Murphy et al.60 | 2017 | PTS | 200-2015 | Wallstent | 71 | 2 minor bleeds |
| Partovi et al.61 | 2017 | PTS | 2008-2010 | Wallstent | 7 | |
| Ruihua et al.62 | 2017 | PTS | 2013-2014 | Wallstent E-Luminexx | 81 | 46 cases of back pain |
| van Vuuren et al.63 | 2017 | NIVL PTS | 2009-2016 | Sinus obliquus Sinus venous Sinus XL Sinus XL superflex Veniti vici Venovo Zilver vena | 369 | 34 cases of stent compression, 32 minor bleeds, 12 major bleeds, 2 arterial injuries and 1 stent fracture |
| Black et al.64 | 2018 | PTS | 2014-2016 | Sinus XL Vici venovo | 88 | 1 Spinal bleed, 5 stent compressions, 3 stent fractures and 1 stent maldeployed |
| Grøtta et al.65 | 2018 | PTS | 2009-2016 | Wallstent | 39 | |
| Liu et al.66 | 2018 | DVT | 2014-2016 | Wallstent | 91 | 1 minor bleed |
| Rizvi et al.67 | 2018 | NIVL | 2013-2014 | Wallstent | 210 | |
| Ye et al.68 | 2018 | PTS | 2012-2015 | Wallstent E-Luminexx | 114 | |
| Yu et al.69 | 2018 | DVT | 2009-2014 | SMART | 40 | 2 minor bleeds |
| Avgerinos et al.70 | 2019 | DVT | 2007-2017 | Wallstent Protégé | 72 | 5 minor bleeds and 2 major bleeds |

## Slide 8
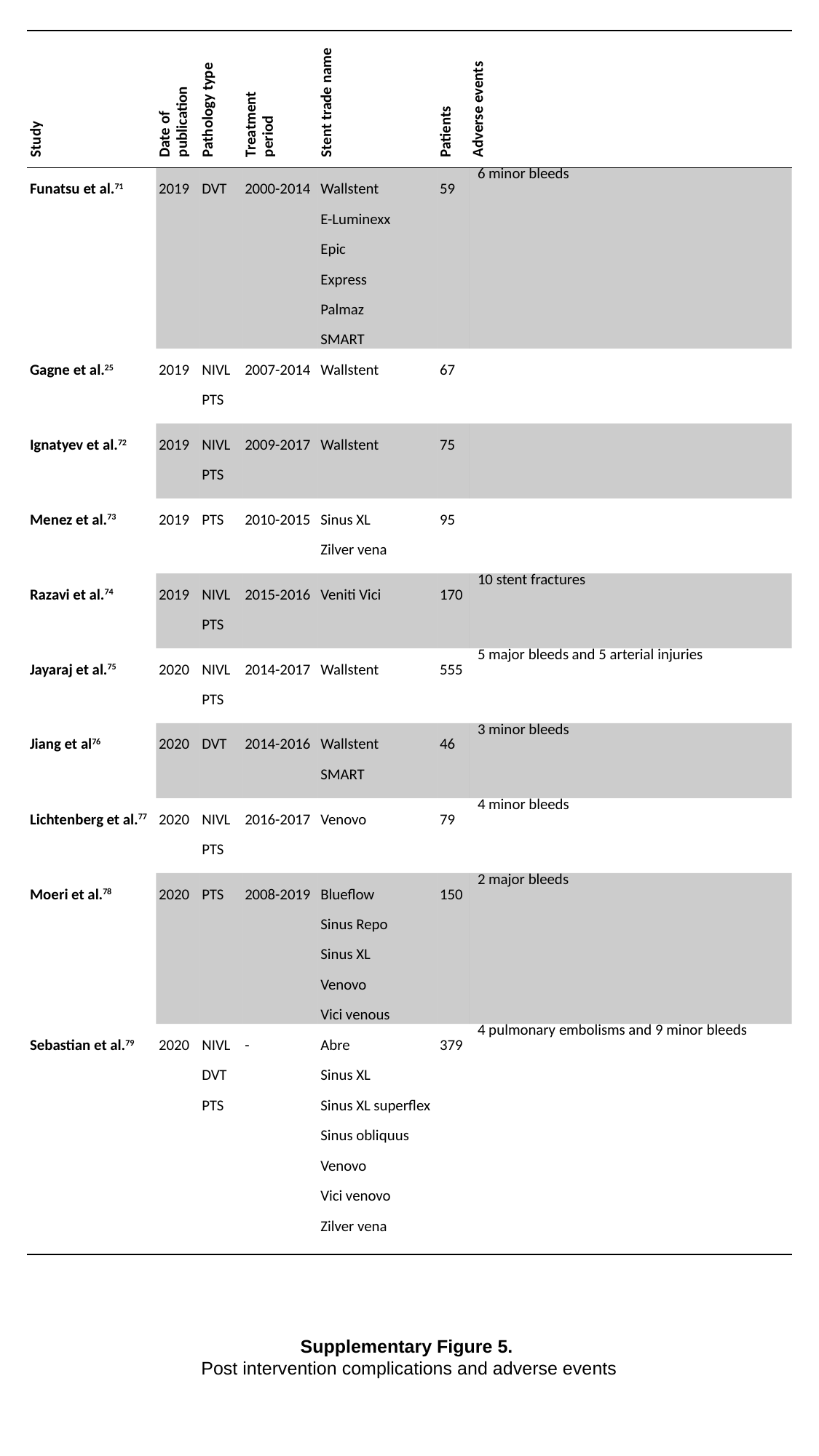

| Study | Date of publication | Pathology type | Treatment period | Stent trade name | Patients | Adverse events |
| --- | --- | --- | --- | --- | --- | --- |
| Funatsu et al.71 | 2019 | DVT | 2000-2014 | Wallstent E-Luminexx Epic Express Palmaz SMART | 59 | 6 minor bleeds |
| Gagne et al.25 | 2019 | NIVL PTS | 2007-2014 | Wallstent | 67 | |
| Ignatyev et al.72 | 2019 | NIVL PTS | 2009-2017 | Wallstent | 75 | |
| Menez et al.73 | 2019 | PTS | 2010-2015 | Sinus XL Zilver vena | 95 | |
| Razavi et al.74 | 2019 | NIVL PTS | 2015-2016 | Veniti Vici | 170 | 10 stent fractures |
| Jayaraj et al.75 | 2020 | NIVL PTS | 2014-2017 | Wallstent | 555 | 5 major bleeds and 5 arterial injuries |
| Jiang et al76 | 2020 | DVT | 2014-2016 | Wallstent SMART | 46 | 3 minor bleeds |
| Lichtenberg et al.77 | 2020 | NIVL PTS | 2016-2017 | Venovo | 79 | 4 minor bleeds |
| Moeri et al.78 | 2020 | PTS | 2008-2019 | Blueflow Sinus Repo Sinus XL Venovo Vici venous | 150 | 2 major bleeds |
| Sebastian et al.79 | 2020 | NIVL DVT PTS | - | Abre Sinus XL Sinus XL superflex Sinus obliquus Venovo Vici venovo Zilver vena | 379 | 4 pulmonary embolisms and 9 minor bleeds |
Supplementary Figure 5.
Post intervention complications and adverse events

## Slide 9
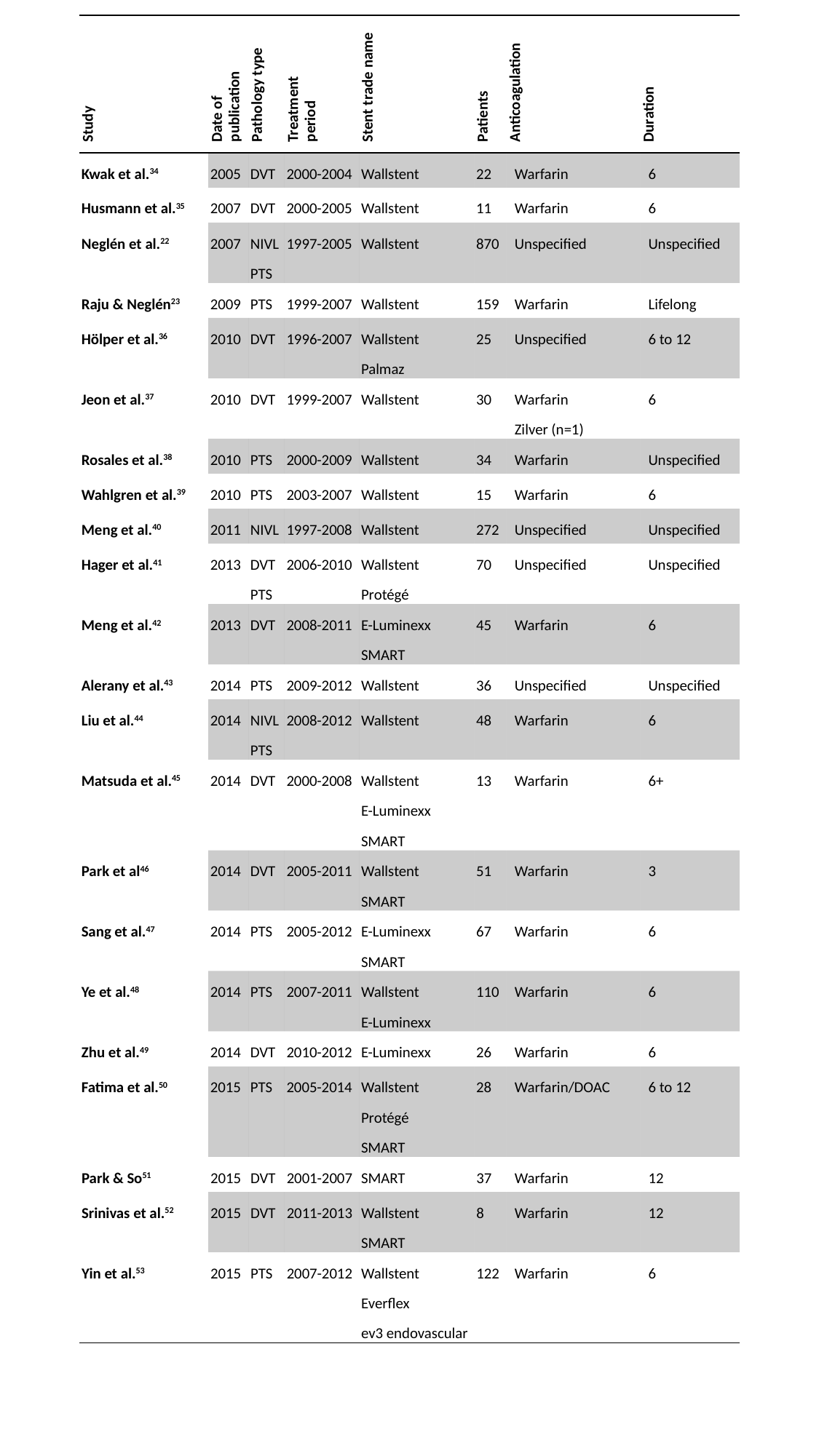

| Study | Date of publication | Pathology type | Treatment period | Stent trade name | Patients | Anticoagulation | Duration |
| --- | --- | --- | --- | --- | --- | --- | --- |
| Kwak et al.34 | 2005 | DVT | 2000-2004 | Wallstent | 22 | Warfarin | 6 |
| Husmann et al.35 | 2007 | DVT | 2000-2005 | Wallstent | 11 | Warfarin | 6 |
| Neglén et al.22 | 2007 | NIVL PTS | 1997-2005 | Wallstent | 870 | Unspecified | Unspecified |
| Raju & Neglén23 | 2009 | PTS | 1999-2007 | Wallstent | 159 | Warfarin | Lifelong |
| Hölper et al.36 | 2010 | DVT | 1996-2007 | Wallstent Palmaz | 25 | Unspecified | 6 to 12 |
| Jeon et al.37 | 2010 | DVT | 1999-2007 | Wallstent | 30 | Warfarin Zilver (n=1) | 6 |
| Rosales et al.38 | 2010 | PTS | 2000-2009 | Wallstent | 34 | Warfarin | Unspecified |
| Wahlgren et al.39 | 2010 | PTS | 2003-2007 | Wallstent | 15 | Warfarin | 6 |
| Meng et al.40 | 2011 | NIVL | 1997-2008 | Wallstent | 272 | Unspecified | Unspecified |
| Hager et al.41 | 2013 | DVT PTS | 2006-2010 | Wallstent Protégé | 70 | Unspecified | Unspecified |
| Meng et al.42 | 2013 | DVT | 2008-2011 | E-Luminexx SMART | 45 | Warfarin | 6 |
| Alerany et al.43 | 2014 | PTS | 2009-2012 | Wallstent | 36 | Unspecified | Unspecified |
| Liu et al.44 | 2014 | NIVL PTS | 2008-2012 | Wallstent | 48 | Warfarin | 6 |
| Matsuda et al.45 | 2014 | DVT | 2000-2008 | Wallstent E-Luminexx SMART | 13 | Warfarin | 6+ |
| Park et al46 | 2014 | DVT | 2005-2011 | Wallstent SMART | 51 | Warfarin | 3 |
| Sang et al.47 | 2014 | PTS | 2005-2012 | E-Luminexx SMART | 67 | Warfarin | 6 |
| Ye et al.48 | 2014 | PTS | 2007-2011 | Wallstent E-Luminexx | 110 | Warfarin | 6 |
| Zhu et al.49 | 2014 | DVT | 2010-2012 | E-Luminexx | 26 | Warfarin | 6 |
| Fatima et al.50 | 2015 | PTS | 2005-2014 | Wallstent Protégé SMART | 28 | Warfarin/DOAC | 6 to 12 |
| Park & So51 | 2015 | DVT | 2001-2007 | SMART | 37 | Warfarin | 12 |
| Srinivas et al.52 | 2015 | DVT | 2011-2013 | Wallstent SMART | 8 | Warfarin | 12 |
| Yin et al.53 | 2015 | PTS | 2007-2012 | Wallstent Everflex ev3 endovascular | 122 | Warfarin | 6 |

## Slide 10
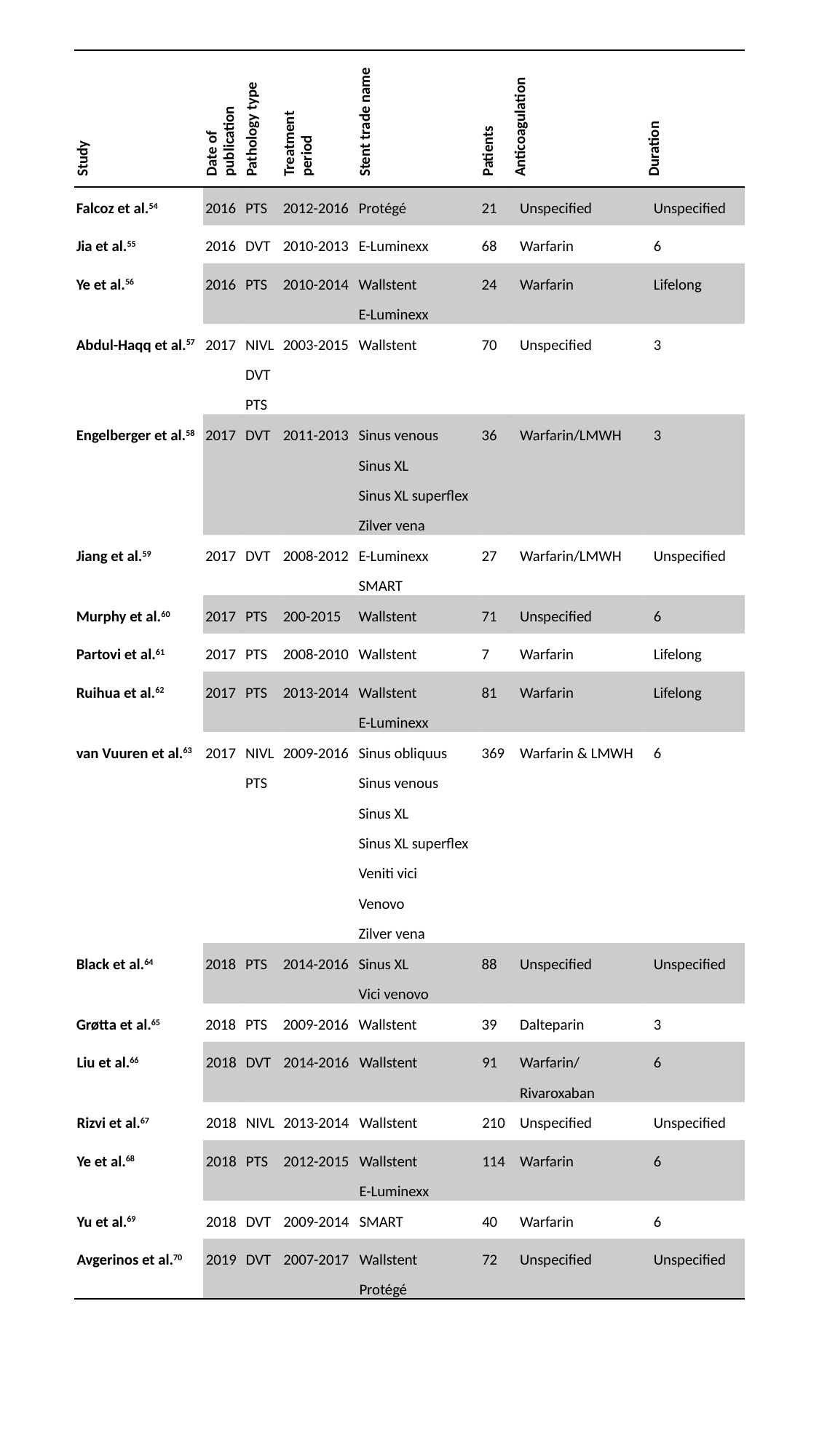

| Study | Date of publication | Pathology type | Treatment period | Stent trade name | Patients | Anticoagulation | Duration |
| --- | --- | --- | --- | --- | --- | --- | --- |
| Falcoz et al.54 | 2016 | PTS | 2012-2016 | Protégé | 21 | Unspecified | Unspecified |
| Jia et al.55 | 2016 | DVT | 2010-2013 | E-Luminexx | 68 | Warfarin | 6 |
| Ye et al.56 | 2016 | PTS | 2010-2014 | Wallstent E-Luminexx | 24 | Warfarin | Lifelong |
| Abdul-Haqq et al.57 | 2017 | NIVL DVT PTS | 2003-2015 | Wallstent | 70 | Unspecified | 3 |
| Engelberger et al.58 | 2017 | DVT | 2011-2013 | Sinus venous Sinus XL Sinus XL superflex Zilver vena | 36 | Warfarin/LMWH | 3 |
| Jiang et al.59 | 2017 | DVT | 2008-2012 | E-Luminexx SMART | 27 | Warfarin/LMWH | Unspecified |
| Murphy et al.60 | 2017 | PTS | 200-2015 | Wallstent | 71 | Unspecified | 6 |
| Partovi et al.61 | 2017 | PTS | 2008-2010 | Wallstent | 7 | Warfarin | Lifelong |
| Ruihua et al.62 | 2017 | PTS | 2013-2014 | Wallstent E-Luminexx | 81 | Warfarin | Lifelong |
| van Vuuren et al.63 | 2017 | NIVL PTS | 2009-2016 | Sinus obliquus Sinus venous Sinus XL Sinus XL superflex Veniti vici Venovo Zilver vena | 369 | Warfarin & LMWH | 6 |
| Black et al.64 | 2018 | PTS | 2014-2016 | Sinus XL Vici venovo | 88 | Unspecified | Unspecified |
| Grøtta et al.65 | 2018 | PTS | 2009-2016 | Wallstent | 39 | Dalteparin | 3 |
| Liu et al.66 | 2018 | DVT | 2014-2016 | Wallstent | 91 | Warfarin/ Rivaroxaban | 6 |
| Rizvi et al.67 | 2018 | NIVL | 2013-2014 | Wallstent | 210 | Unspecified | Unspecified |
| Ye et al.68 | 2018 | PTS | 2012-2015 | Wallstent E-Luminexx | 114 | Warfarin | 6 |
| Yu et al.69 | 2018 | DVT | 2009-2014 | SMART | 40 | Warfarin | 6 |
| Avgerinos et al.70 | 2019 | DVT | 2007-2017 | Wallstent Protégé | 72 | Unspecified | Unspecified |

## Slide 11
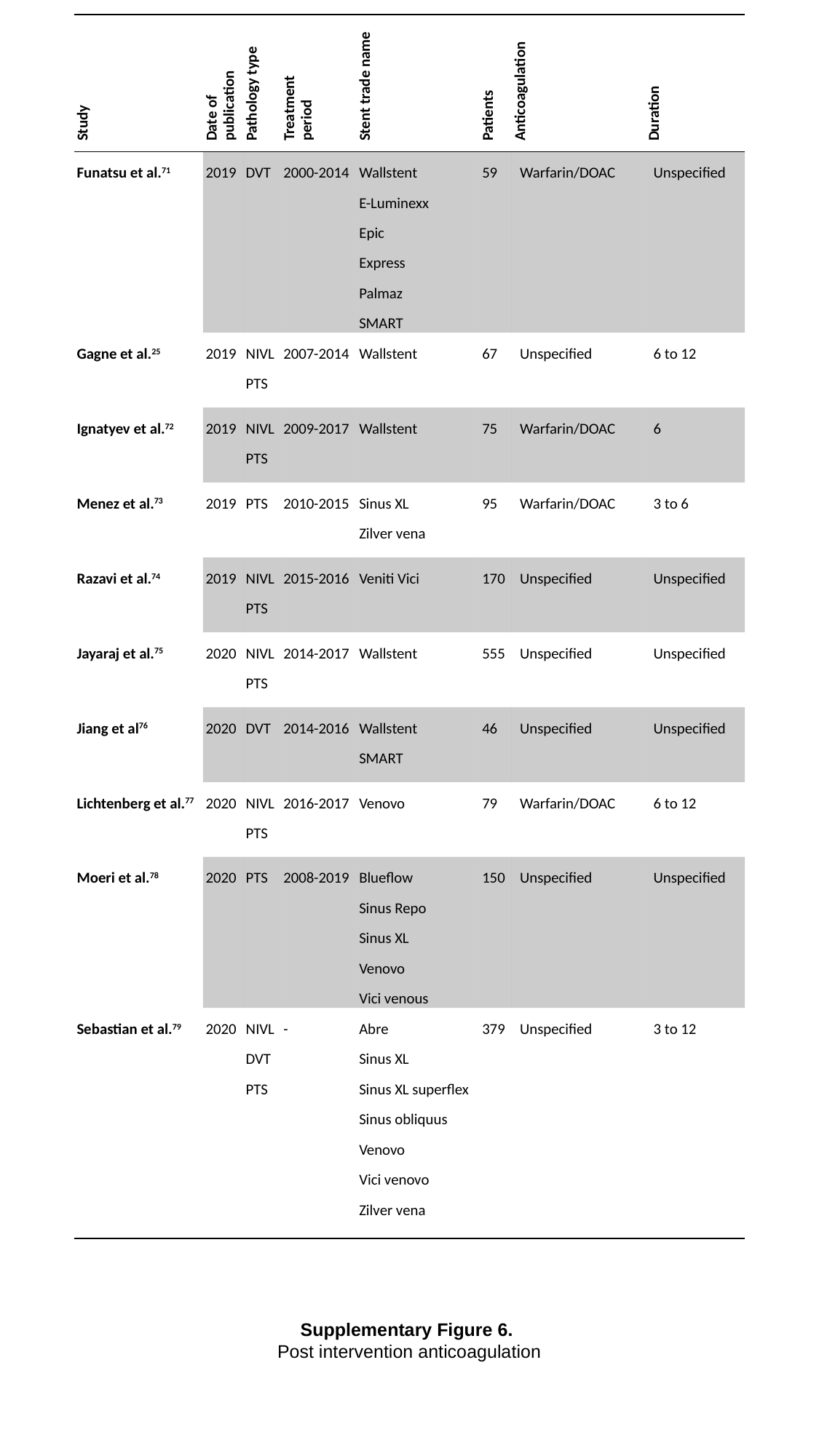

| Study | Date of publication | Pathology type | Treatment period | Stent trade name | Patients | Anticoagulation | Duration |
| --- | --- | --- | --- | --- | --- | --- | --- |
| Funatsu et al.71 | 2019 | DVT | 2000-2014 | Wallstent E-Luminexx Epic Express Palmaz SMART | 59 | Warfarin/DOAC | Unspecified |
| Gagne et al.25 | 2019 | NIVL PTS | 2007-2014 | Wallstent | 67 | Unspecified | 6 to 12 |
| Ignatyev et al.72 | 2019 | NIVL PTS | 2009-2017 | Wallstent | 75 | Warfarin/DOAC | 6 |
| Menez et al.73 | 2019 | PTS | 2010-2015 | Sinus XL Zilver vena | 95 | Warfarin/DOAC | 3 to 6 |
| Razavi et al.74 | 2019 | NIVL PTS | 2015-2016 | Veniti Vici | 170 | Unspecified | Unspecified |
| Jayaraj et al.75 | 2020 | NIVL PTS | 2014-2017 | Wallstent | 555 | Unspecified | Unspecified |
| Jiang et al76 | 2020 | DVT | 2014-2016 | Wallstent SMART | 46 | Unspecified | Unspecified |
| Lichtenberg et al.77 | 2020 | NIVL PTS | 2016-2017 | Venovo | 79 | Warfarin/DOAC | 6 to 12 |
| Moeri et al.78 | 2020 | PTS | 2008-2019 | Blueflow Sinus Repo Sinus XL Venovo Vici venous | 150 | Unspecified | Unspecified |
| Sebastian et al.79 | 2020 | NIVL DVT PTS | - | Abre Sinus XL Sinus XL superflex Sinus obliquus Venovo Vici venovo Zilver vena | 379 | Unspecified | 3 to 12 |
Supplementary Figure 6.
Post intervention anticoagulation
